# Use of Heat Stress Responsive Gene Expression Levels for Early Selection of Heat Tolerant Cabbage (*Brassica oleracea* L.)

**DOI:** 10.3390/ijms140611871

**Published:** 2013-06-04

**Authors:** Hyun Ji Park, Won Yong Jung, Sang Sook Lee, Jun Ho Song, Suk-Yoon Kwon, HyeRan Kim, ChulWook Kim, Jun Cheul Ahn, Hye Sun Cho

**Affiliations:** 1 Plant Systems Engineering Research Center, Korea Research Institute of Bioscience and Biotechnology (KRIBB), 125 Gwahangno, Yuseong-gu, Daejeon 305-806, Korea; E-Mails: hotfehj@kribb.re.kr (H.J.P.); jwy95@kribb.re.kr (W.Y.J.); sslee@kribb.re.kr (S.S.L.); sykwon@kribb.re.kr (S.-Y.K.); kimhr@kribb.re.kr (H.K.); 2 Department of Animal Resources Technology, Gyeongnam National University of Science and Technology, Jinju 660-758, Korea; E-Mail: cwkim@gntech.ac.kr; 3 Asia Seed Company, 447-2, Inhwang-Ri, Janghowon-Eup, Ichen 467-906, Korea; E-Mail: cabrojhs@yahoo.co.kr; 4 Department of Pharmacology, Medical Sciences, Seonam University, Kwangchi-dong, Namwon 590-711, Korea

**Keywords:** heat shock proteins, heat shock transcription factors, heat tolerance, Hsp70, GRAS, cabbage breeding, early selection

## Abstract

Cabbage is a relatively robust vegetable at low temperatures. However, at high temperatures, cabbage has disadvantages, such as reduced disease tolerance and lower yields. Thus, selection of heat-tolerant cabbage is an important goal in cabbage breeding. Easier or faster selection of superior varieties of cabbage, which are tolerant to heat and disease and have improved taste and quality, can be achieved with molecular and biological methods. We compared heat-responsive gene expression between a heat-tolerant cabbage line (HTCL), “HO”, and a heat-sensitive cabbage line (HSCL), “JK”, by Genechip assay. Expression levels of specific heat stress-related genes were increased in response to high-temperature stress, according to Genechip assays. We performed quantitative RT-PCR (qRT-PCR) to compare expression levels of these heat stress-related genes in four HTCLs and four HSCLs. Transcript levels for heat shock protein *BoHsp70* and transcription factor *BoGRAS* (*SCL13)* were more strongly expressed only in all HTCLs compared to all HSCLs, showing much lower level expressions at the young plant stage under heat stress (HS). Thus, we suggest that expression levels of these genes may be early selection markers for HTCLs in cabbage breeding. In addition, several genes that are involved in the secondary metabolite pathway were differentially regulated in HTCL and HSCL exposed to heat stress.

## 1. Introduction

Cabbage (*Brassica oleracea* L.) is a low-calorie leafy vegetable that is high in vitamin C, minerals and dietary fiber [1]. However, cabbage cultivation is vulnerable to high temperatures. Thus, cultivation is typically restricted to the highland areas of the tropics or subtropics. Breeding of heat-tolerant cabbage varieties has been a key focus of cabbage seed companies for many years [2]. In addition to heat tolerance, another research interest has been to develop improved cabbage varieties that are resistant to insects or disease and have various tastes or colors. Therefore, discovering a way to select heat-tolerant lines quickly and quantitatively will contribute to the breeding and development of new heat-tolerant cabbage varieties.

Exposure of plants to above-optimal growth temperatures affects the enzymatic activities required for many essential metabolic processes, including photosynthesis, carbon fixation and development. Thus, plants defend against heat-induced damage by retaining components required for maintenance of cellular homeostasis. In particular, molecular chaperones play critical roles in the cellular environment by helping to ensure that proteins are folded and assembled correctly. Many molecular chaperones function as heat shock proteins (Hsps) [3]. Hsps and other stress proteins protect cells against the deleterious effects of stress [4–9]. In some cells, Hsps are constitutively expressed. In other cells, Hsp expression is regulated by the cell cycle or development [10,11].

The five distinct classes of Hsps according to molecular weights are Hsp100s, Hsp90s, Hsp70s, Hsp60s and small Hsps (sHsps). Hsp60s are found in prokaryotes and in eukaryotic mitochondria and plastids. Hsp60s help to ensure that newly made proteins are correctly assembled [12,13]. Hsp70s are highly conserved, with at least 50% amino acid homology retained through evolution at the *N-*terminal ATPase domain and *C*-terminal peptide binding domain [14]. Hsp70s are strongly induced by heat shock and other cellular stresses. Some Hsp70s are constitutively expressed and have essential functions that are not related to stress [15]. Plants that overexpress the *Hsp70* genes are tolerant to heat and have increased resistance to environmental stressors [15–17]. In addition to functioning as general chaperones, Hsp70s also regulate expression of stress-associated genes [18]. In contrast to many Hsps, most Hsp90 substrates are signaling proteins, including receptors for steroid hormones and kinases. Thus, although Hsp90 plays an important role in protein folding, it also has functions in signaling, cell cycle regulation, protein turnover and localization, morphology and the cellular response to stress [12,19–21]. Hsp100s are members of the large AAA ATPase superfamily and have diverse functions [22,23]. Hsp100s are important for protein disaggregation and/or degradation. Although constant expression of Hsp100s is often observed in plants, developmental processes or environmental stressors may also regulate expression [24–27]. The low-molecular weight (12–40 kDa) sHsps are the most abundant group of Hsps and are uniquely expressed in higher plants. Although sHsps do not directly assist with protein folding, they do help facilitate protein folding by other ATP-dependent chaperones, probably through hydrophobic interactions with non-native proteins [28–30]. The diversification of plant sHsps might be related to molecular adaptations to stress conditions that are unique to plants [15].

Heat stress transcription factors (Hsfs) are the central regulators of the heat shock (HS) stress response [31]. The overall basic structures and consensus DNA-binding sites of Hsfs are conserved from yeast to humans [32]. Plants possess large families of genes that encode Hsfs. For example, *Arabidopsis* plants have 21 genes that encode Hsfs, and rice plants have 23 Hsf genes. In contrast, yeast have one Hsf gene, and humans have three Hsf genes [6,31]. In addition, 28 *Poplus trichocarpa* Hsfs and 16 *Medicago truncatula* Hsfs were identified through bioinformatics analyses. Seventeen Hsfs have been identified in tomato from expressed sequence tags (ESTs) [33,34]. There are three groups of plant Hsfs (A, B and C). These groups are based on the length of the area between the DNA-binding domain and the hydrophobic coiled-coil region of the Hsf protein. The consensus Hsf binding sequence, “nGAAnnTCCn”, is located in the promoter region of many defense genes. Thus, plant Hsfs control expression of genes that have overlapping or flexible functions in the response of plants to various environmental stressors [35].

In this report, to provide a way to quickly determine cabbage heat tolerance at the young stage and *in vitro*, expression levels of heat stress and secondary metabolism-related genes were examined in young heat-tolerant cabbage lines (HTCLs) and young heat-sensitive cabbage lines (HSCLs), which were grown in normal conditions (NS) or at a high temperature (HS), reexamined to distinguish heat shock tolerance in the early stage of four HTCLs and four HSCLs in the heat stress condition using qRT-PCR. In addition, expression levels of secondary metabolism-related genes between an HTCL and HSCL were evaluated.

## 2. Results

### 2.1. The Phenotypic Differences between HTCLs and HSCLs at a High Temperature

**Figure 1:**
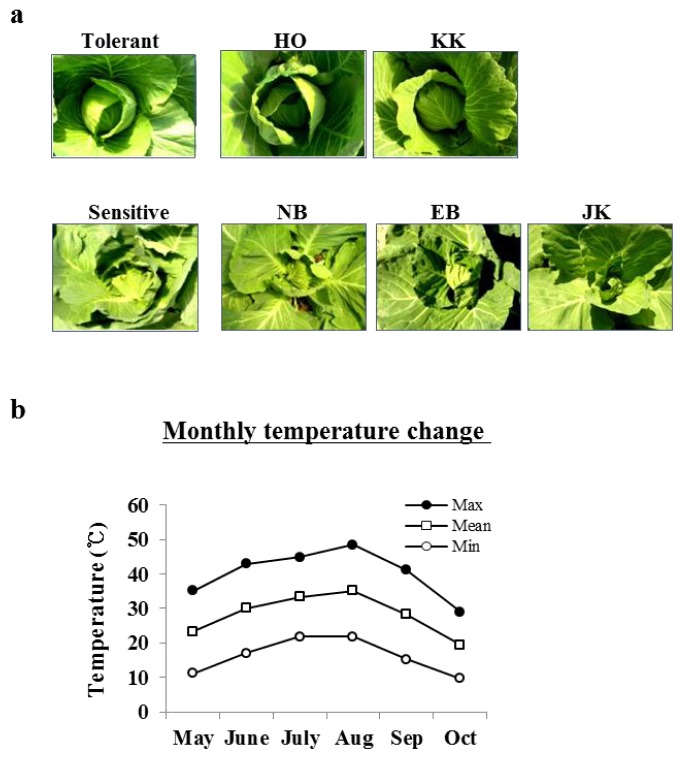
Heat stress phenotype of cabbage head formation after summer season. These photos were taken after the summer season had passed. (**a**) Phenotypes of inbred lines HO, KK, NB, EB and JK (Asia Seed Company). The HO and KK lines showed heat tolerance, whereas the NB, EB and JK lines showed heat-sensitive phenotypes in the field condition. A heat-tolerant cultivar and a heat-sensitive cultivar were used as a positive or negative control of heat shock stress in the field condition. Seeds were sown in May, and young plants were transplanted in June. Photos were taken at the end of October; (**b**) Temperature changes during cultivation of cabbages in a vinyl house in the field condition are shown. Max, maximum temperature; Mean, average temperature; Min, minimum temperature.

Cabbage head formation depends on a significant genotype-environment interaction [36]. Thus, the main phenotypic difference that we examined between HTCLs and HSCLs was the ability to form cabbage heads at a high temperature. Inbred HTCLs started to form heads during the vegetative growth period, whereas inbred HSCLs did not (Figure 1a). The HTCLs “HO” and “KK” and the HSCLs “NB”, “EB” and “JK” were developed by the Asia Seed Company and cultivated from May 2011 to October 2011 at a company greenhouse in the Gyeonggi-Do area. Photos were taken on October 10, 2011. Although the leaf position at which head formation started (LPH) varied between the two HTCLs, both lines started normal head formation (data not presented). In contrast, the three HSCLs showed poor head formation. Temperature changes in the greenhouse during the growth period for the cabbage lines are shown in Figure 1b. The maximum temperature at midday exceeded 40 °C from June and reached a maximum of 48.5 °C in August.

### 2.2. Heat Stress-Responsive Transcriptome between HTCL “HO” and HSCL “JK” by Brassica Microarray

Expression analyses were performed on two inbred lines (“HO” for HTCL and “JK” for HSCL) at an early developmental stage (two-week-old, young plants) in HS and NS conditions on an Agilent Brassica GE 2 × 105k microarray. In the HTCL “HO” line, 500 and 453 transcripts were differently up- and down-regulated in the HS and NS condition, respectively. In the HSCL “JK” line, 703 and 834 transcripts were identified as being up- and down-regulated in the HS and NS condition, respectively (data not shown).

### 2.3. Functional Categories of Heat Stress Response Genes in Cabbage

**Figure 2:**
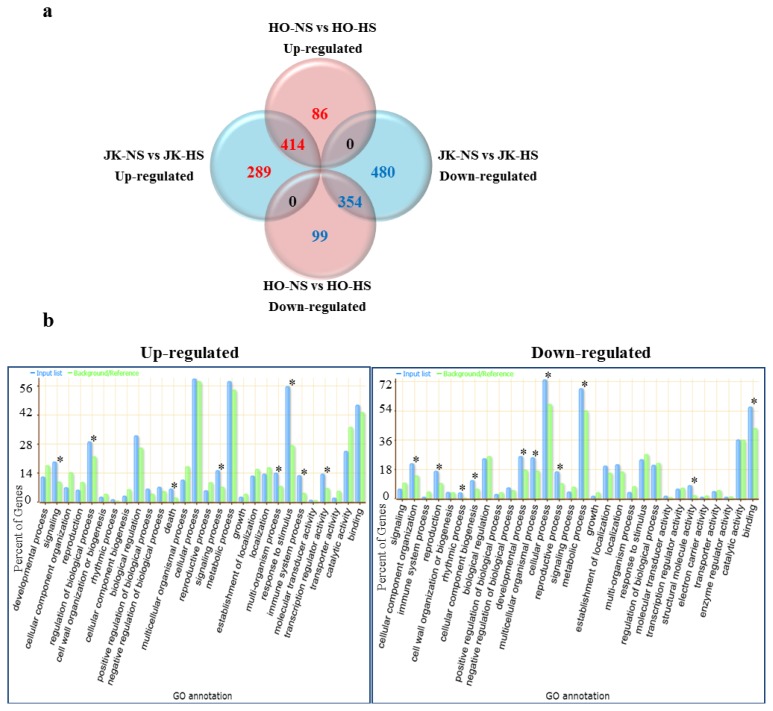
Microarray analysis of transcripts that are differentially expressed between heat stress-tolerant and heat-sensitive cabbage inbred lines exposed to heat stress. (**a**) Venn diagram showing differentially expressed transcripts of the two cabbage lines in response to heat stress and no heat stress conditions. HO-NS, heat stress-tolerant cabbage “HO” inbred line—no heat stress; HO-HS, heat stress-tolerant cabbage “HO” inbred line—heat stress; JK-NS, heat stress-sensitive cabbage “JK” inbred line—no heat stress; JK-HS, heat stress-sensitive cabbage “JK” inbred line—heat stress. The numbers of upregulated genes are shown in red, whereas the numbers of downregulated genes are shown in blue (fold change ≥2 and ≤−2, *p* ≤ 0.05); (**b**) Functional analysis of differentially expressed transcripts during heat stress or no heat stress in two cabbage lines. This chart shows GO terms for biological processes and molecular functions for 414 upregulated genes and 354 downregulated genes in tolerant and sensitive cabbages (fold change ≥2 and ≤−2, *p* ≤ 0.05). * Over-represented GO terms in the categories.

Venn diagram analysis identified 414 and 354 transcripts with significant changes in expression in response to heat stress in HTCL and HSCL, respectively (Figure 2a). We further characterized genes that have significant GO terms (*p* ≤ 0.05) in biological processes and molecular functions with the agriGO web-based GO analysis toolkit [37] and *Arabidopsis* orthologs. The GO annotation of upregulated genes in both cabbage lines in the HS condition revealed that 414 genes were significantly assigned with GO terms for signaling, regulation of biological processes, immune system processes, death, multi-organism processes, response to stimuli and regulation of transcriptional activity (Figure 2b). Among the GO terms associated with response to stimuli in biological processes, the most significant categories were response to chemical stimuli, chitin, heat, light intensity and stress. Transcriptional regulator activity was the only significant molecular function category. Many of the genes were heat stress-responsive genes, like HSPs, and showed a high level of expression. Similarly, 354 genes were downregulated in the HS condition. GO terms were assigned for genes involved in development, multicellular organisms, reproduction organization of cellular components, rhythmic processes, cellular processes, metabolic processes, cellular component biogenesis and structural molecule activity and binding (Figure 2b). The GO terms for biological process included a large range of GO categories, which were other cellular processes, other metabolic processes, developmental processes, cell organization and biogenesis, DNA or RNA metabolism, one-carbon metabolic processes and protein metabolism. GO terms for the molecular function indicated that the genes represented GO categories, such as other binding, DNA or RNA binding, hydrolase activity, nucleotide binding, other enzyme activity, transferase activity, protein binding, structural molecular activity and others. Many genes encoded ribosomal proteins, binding proteins and enzyme activity-related genes, all of which showed low expression patterns.

### 2.4. Heat-Responsive HSPs and HSFs from Genechip Analysis

**Figure 3:**
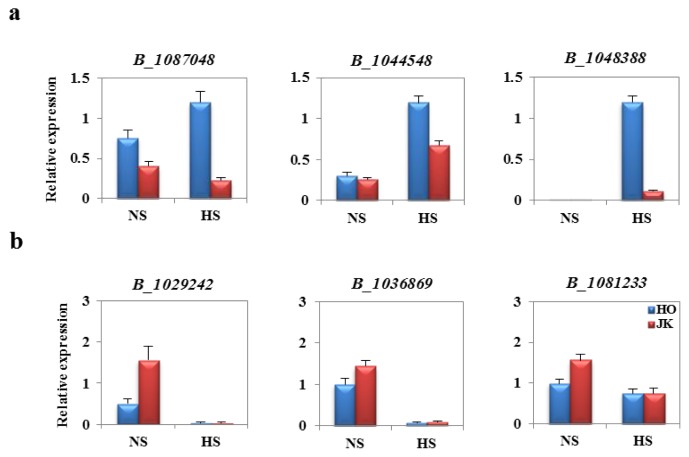
Validation of microarray data by quantitative real-time PCR. Transcript levels of a subset of up- and down-regulated genes from microarray data. Two-week-old plants were exposed to heat stress at 42 °C for 4 h (HS) or incubated at 24 °C (NS). (**a**) Transcript levels of upregulated genes in the heat stress condition in HO and JK lines. (*B_1087048*, *Putative splicing factor; B_1044548*, *GRAS family transcription factor; B_1048388*, *70 kDa heat shock protein*). HO is a heat-tolerant cabbage inbred line; JK is a heat-sensitive cabbage inbred line; (**b**) Transcripts levels of downregulated genes in the heat stress condition in HO and JK lines. (*B_1029242*, *Myb-related transcription factor; B_1036869*, *Pectate lyase 18 precursor; B_1081233*, *Hydrogen-transporting ATP synthase*). Expression levels were normalized to *BoActin.*

HSPs play important roles in response to environmental conditions and in various developmental processes. To identify heat stress-responsive *Hsp* genes, we identified *Hsp* genes that showed very high expression patterns in inbred cabbage lines by microarray analysis. Only 40 genes (24 *Arabidopsis* orthologs) were identified from the 103,748 *Brassica* unigenes (Table 1). Thirty-eight genes were upregulated in both lines. Two genes were downregulated in both lines. These 40 *Hsp* genes were classified as *hsp100*, *hsp90*, *hsp70*, *small-hsp* and unclassified. Seven HSP genes were identified as being differentially upregulated with FC ≥ 50, and 12 genes had greater than 10-fold changes in expression levels. Furthermore, we found that most of HSPs were upregulated by HS. Eleven *HSF* genes (five *Arabidopsis* orthologs) were identified from the unigenes (Table 2) and were significantly upregulated by heat stress response. These genes were induced by more than ten-fold in HS compared to NS in both HTCLs and HSCLs. However, *BoHsfB1*, *BoHdfA7b* and *BoHsfA4c* were excluded from this study, because their expression levels did not change more than four-fold (data not shown). Before identifying heat shock-related genes, we carried out qRT-PCR analyses to verify the Genechip data. The first group of upregulated genes in the HS condition, B_1087048, B_1044548 and B_1048388 (probe from *Brassica* unigene), were increased in HS compared to NS, whereas the downregulated genes in the HS condition, B_102942, B_1036869 and B_1081233, were decreased in HS compared to NS (Figure 3a,b). These results demonstrate that Genechip analysis can select heat shock response genes.

### 2.5. Heat Shock Phenotypes of HTCLs and HSCLs *in Vitro*

**Figure 4:**
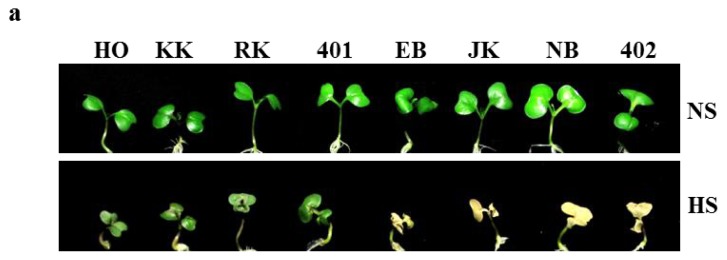

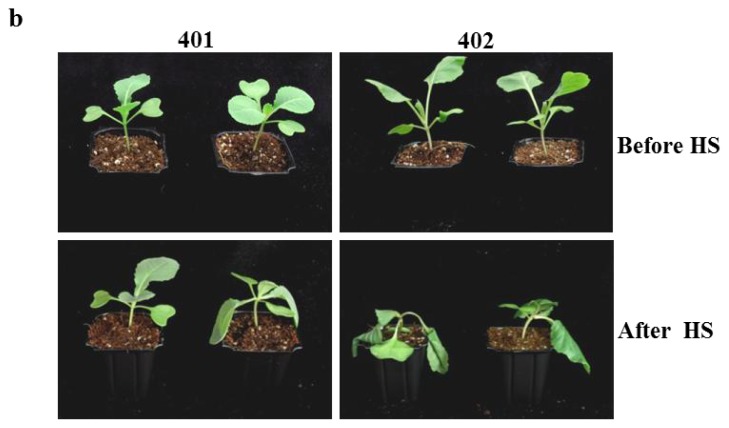
Heat stress phenotypes of heat-tolerant or heat-sensitive inbred cabbages. (**a**) Heat stress phenotypes of seedling stage cabbages. HO, KK, RK and 401 are heat-tolerant inbred lines. EB, JK, NB and 402 are heat-sensitive inbred lines. One-week-old seedlings were grown on MS media containing 1% sucrose at normal conditions (24 °C). Heat stress was performed in a 42 °C incubator for 5 h with the recovery at 24 °C, normal condition (HS). In contrast, non-heat-treated seedlings were grown continuously at 24 °C (NS). Photos were taken on the fourth day after heat stress exposure; (**b**) Heat stress phenotypes of young heat-tolerant and heat-sensitive inbred lines. Two-week-old, young plants were grown on sterilized soil at normal growth conditions (24 °C). Heat stress was performed in a 42 °C incubator for 4 h with recovery in normal conditions. Photos were taken before heat stress treatment and on the third day after heat stress treatment.

To evaluate the phenotype of HTCLs, “HO”, “KK”, “RK” and “401”, and HSCLs, “EB”, “JK”, “NB” and “402”, one-week-old seedlings were treated in a 42 °C incubator for 5 h followed by recovery to normal temperature (24 °C). All of the HTCLs showed obvious heat-tolerant phenotypes in the HS stress condition compared to all of the HSCLs (Figure 4a). However, no differences were observed between HTCLs and HSCLs at the seedling stage in the NS condition. Cotyledon expansion and emergence of new leaves were normal and not much different in all eight plant lines at 24 °C. However, HS exposure had a considerable impact on seedlings. The effects revealed significant differences between HTCLs and HSCLs. The green cotyledon was maintained in both NS and HS conditions. However, the HSCLs, “EB”, “JK”, “NB” and “402”, showed a necrotic phenotype with yellowish stems and leaves and eventually died (Figure 4a) at the HS condition. Next, in soil-planted conditions, two-week-old, young HTCL “401” and HSCL “402” plants were heat-shocked at 42 °C for 4 h and then recovered at 24 °C. HTCL “401” showed minor growth retardation on the third day after heat stress, but HSCL “402” showed severe leaf-bending, wilting and senescence at the HS condition. These data are consistent with the results presented in Figure 1a (Figure 4b). Similar to “401” and “402”, differences in heat stress phenotypes were observed between other young HTCLs and HSCLs grown on soil conditions (data not shown). From these results, we confirmed that there is a correlation between field test results for heat shock phenotype and *in vitro* culture conditions in HTCLs and HSCLs. Thus, these inbred lines provide resource material for studying the heat stress-tolerant trait in cabbage crops. We compared the expression levels of heat shock response genes in these inbred lines.

### 2.6. Analysis of Fold Change in Expression of Heat Stress-Related Genes between HTCLs and HSCLs

**Figure 5:**
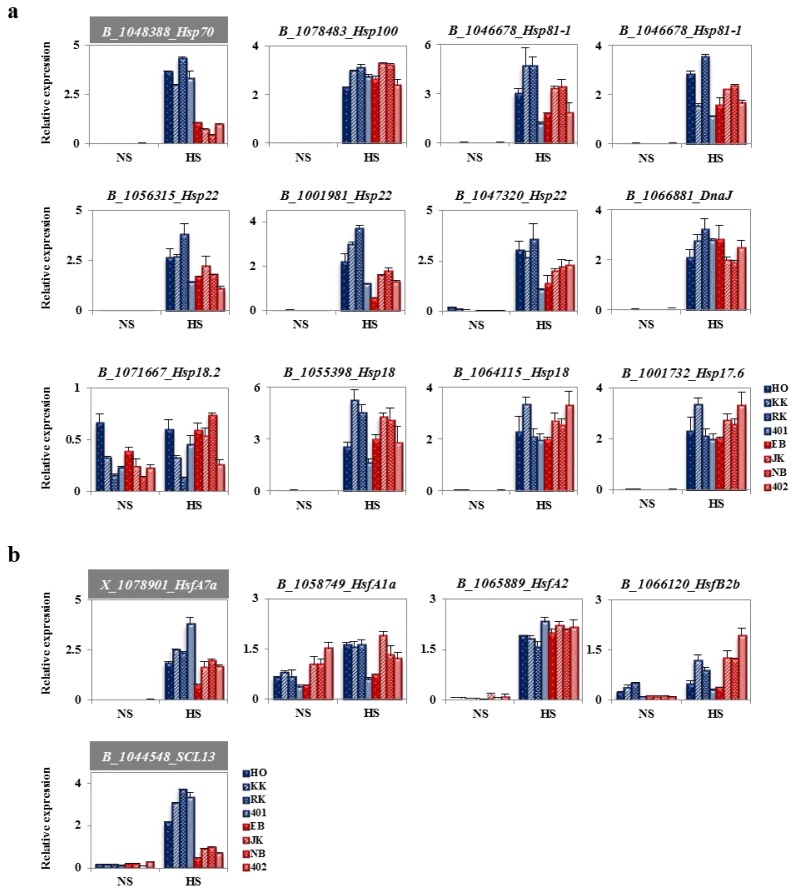
Analysis of fold change in expression of heat shock proteins and heat shock transcription factors in heat-tolerant and heat-sensitive cabbage lines. (**a**) Comparative change in fold expression of heat shock proteins in heat-tolerant cabbage lines (HTCLs) and heat-sensitive cabbage lines (HSCLs)*. BoHsp70*, *BoHsp100*, two *BoHsp81s*, three *BoHsp22s*, two *BoHsp18s*, *BoHsp17.6* and *Putative BoHsp*; (**b**) Comparative change in fold expression of heat shock transcription factors in HTCLs and HSCLs. *BoHsfA1a*, *BoHsfA7a*, *BoHsfA2*, *BoHsfB2b* and *BoSCL13*. HO, KK, RK and 401 are heat-tolerant cabbage inbred lines. EB, JK, NB and 402 are heat-sensitive cabbage inbred lines. Two-week old plants were exposed to heat stress at 42 °C for 4 h (HS) or incubated at 24 °C (NS). Expression levels were normalized to *BoActin*.

Heat-shock proteins play crucial roles in protecting cells against stress. They also function in developmental processes in the NS condition and as molecular chaperones in heat stress [14,20]. HSPs are highly conserved amongst organisms. In the previous Genechip analysis, a large number of HSP genes were upregulated at the HS condition in both HTCL and HSCL. In addition, several Hsps were highly expressed in inbred heat-tolerant Chinese cabbage lines [38]. Thus, to compare typical heat-induced expression of HSPs between four HTCLs and four HSCLs, qRT-PCR analyses were performed at NS and HS conditions in two-week-old, young plants. As a result, expression of *BoHsp100*, *BoHsp81s*, *BoHsp70*, *BoHsp22s*, *BoHsp18.2*, *BoHsp18s*, *BoHsp17.6* and putative *BoHsp* (*BoDnaJ*) were evaluated in the HTCLs and HSCLs (Figure 5a). Most *BoHsps* were barely expressed at the NS condition, whereas they were greatly induced at the HS condition in all HTCLs and HSCLs. The exception was *BoHsp18.2*, which showed significant expression, even in the absence of stress. The transcript levels of *BoHSP100* in the HTCLs and HSCLs were dramatically induced at HS compared to NS, but there was no difference between HTCLs and HSCLs. Transcript levels of *BoHSP81s* were tested with two different primers against two *BoHsp81* genes. Although HTCLs and HSCLs showed some differences in expression of one of the *BoHsp81* (B_1046678) at the HS condition, this difference did not extend to all of the HTCLs or HSCLs. In the case of *BoHsp22*, three different cabbage genes were reexamined by qRT-PCR analysis. However, no clear information was gained, due to some inconsistencies between lines. Nevertheless, three *BoHsp22s* showed positive results, suggesting that this family has a potential impact on the heat-tolerant trait. Expression levels of the putative *BoHsp* (B_1066881_DnaJ), *BoHsp18.2*, *BoHsp17.6* and *BoHsp18*s did not differ between HTCLs and HSCLs at the HS condition. In contrast, the expression pattern of *BoHSP70* was increased by about three-fold in all HTCLs compared to the pattern of expression in HSCLs at the HS condition. Therefore, expression of *BoHSP70* may provide a marker for distinguishing heat-tolerant cabbage lines for breeding.

Many heat-inducible genes are regulated by heat shock transcription factors (Hsfs). Hsfs are distal mediators of the cellular signaling response to chemical and environmental stressors [39]. The *Arabidopsis* genome has a very complex Hsf family with 21 members. Because cabbage is also in the *Brassicaceae* family, it may have a similar number of Hsfs. However, only a limited number of Hsfs have a known role in the heat shock response (HR). Most Hsfs are expressed at very low levels and have not yet been shown to have functions in HR. Therefore, we compared expression patterns of Hsfs with known roles in HR by Genechip analysis (Table 2). Expression patterns of *BoHsfA1a*, *BoHsfA7a*, *BoHsfA2*, *BoHsfB2b* and a *BoGRAS transcription factor*, *BoSCL13*, were analyzed in two-week-old, young HTCLs and HSCLs cultured in NS and HS conditions (Figure 5b). *BoHsfA1a* was constitutively expressed in all HTCLs and HSCLs in both NS and HS conditions. The expression levels were not significantly different in HTCLs and HSCLs at the HS condition, whereas HSCLs showed a slightly increased level relative to HTCLs in the NS condition. *BoHsfA7a* was induced in both HTCLs and HSCLs and showed slightly increased expression in all HTCLs compared to HSCLs at the HS condition. *BoHsfA2* was induced at HS in HTCLs and HSCLs. However, there were no differences in *BoHsfA2* expression between tolerant or sensitive lines, despite an essential role in heat tolerance [40,41]. *BoHsf2b* was increased in response to HS in HTCLs and HSCLs, but the expression pattern was very similar between lines at the NS or HS condition, despite differences among the lines. Transcript levels of *BoSCL13*, a *BoGRAS* family gene, were induced in both HTCLs and HSCLs at HS; however, the increase was approximately three-fold higher in HTCLs compared to HSCLs. In summary, these results suggested that *BoHsp70* and *BoSCL13* might be related to heat tolerance and/or proper head formation of cabbage at high temperature. Further, our data suggested that increased expression of these genes might be a marker that distinguishes heat-tolerant cabbage lines.

### 2.7. The Specificity of BoHsp70 and BoSCL11 for the Heat Tolerance Trait in Cabbage

**Figure 6:**
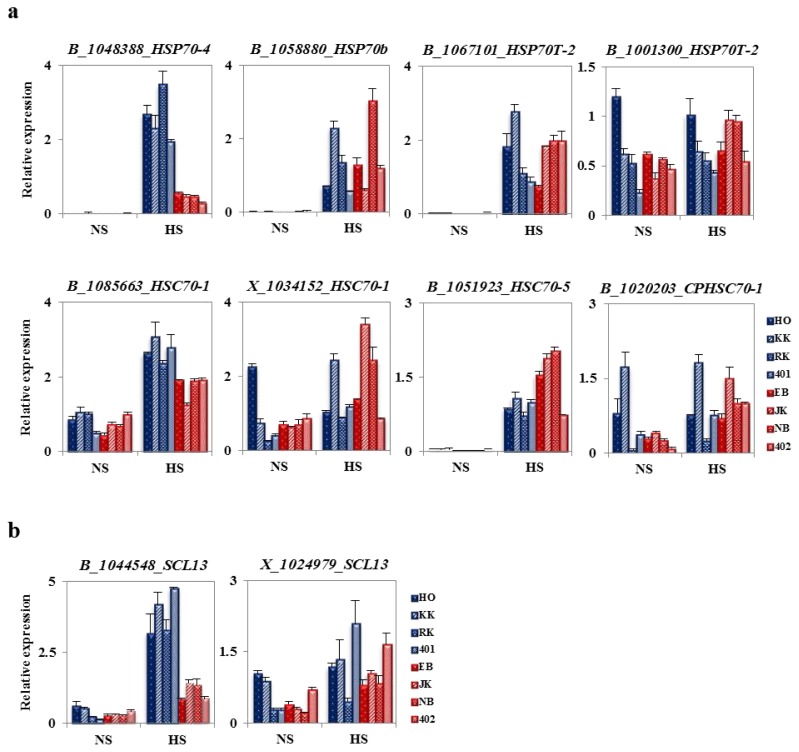
Analysis of fold change in expression of *BoHsp70* and *BoSCL13* family genes in HTCLs and HSCLs. (**a**) Change in expression levels of different *BoHsp70* protein family genes in HTCLs and HSCLs; *BoHsp70*, *BoHsp70bs*, two *BoHsp70T-2s*, two *BoHSC70-1s*, *BoHSC70-5* and *BoCPHSC70-1*; (**b**) Change in expression of two *BoSCL13* genes in HTCLs and HSCLs. HO, KK, RK and 401 are heat-tolerant cabbage inbred lines (HTCLs). EB, JK, NB and 402 are heat-sensitive cabbage inbred lines (HSCLs). Two-week-old plants were heat stress-treated at 42 °C for 4 h (HS) or incubated at 24 °C (NS). Expression levels were normalized to *BoActin*.

To evaluate *BoHsp70* and *BoSCL13* as selective markers for heat tolerance, expression levels of the other genes in this family were analyzed in HTCLs and HSCLs by qRT-PCR. Most *BoHsp70*-family genes were increased clearly at the HS condition compared to the NS condition in both HTCLs and HSCLs. The exception was *BoCPHSC70-1*, a chloroplast Hsp70 gene, which was not induced at the HS condition. Other HSP70 family genes showed no differences in gene expression between HTCLs and HSCLs at NS or HS conditions. *BoHSC70-1* (B_1085663), an Hsp70 cognate gene, showed slightly enhanced expression in all HTCLs (1.25-fold change) compared to HSCLs at the HS condition. Transcript levels of *BoHsp70b*, *BoHsp70T-2s*, *BoHSC70-1s*, *BoHSC70-5* and *BoCPHSC70-1* did not correlate between HTCLs or HSCLs, suggesting that their expression patterns were not useful as selection markers. In the meantime, a primer set against a different region of *BoHsp70* (B_1048388) confirmed differential expression between HTCLs and HSCLs and the potential of this gene as a selective marker (Figure 6a). Because there were two kinds of *BoSCL13* genes on the Genechip analysis, qRT-PCR was performed to determine which of the two genes is specific for the heat stress-tolerant trait. Only *BoSCL13* (B_1044548) was highly expressed in all HTCLs, but not in HSCLs at the HS condition (Figure 6b), whereas another *BoSCL13* (X_1024979) showed no difference between HTCLs and HSCLs. Further, it showed irregular expression patterns among lines. High *BoSCL13* (B_1044548) expression was also confirmed by qRT-PCR with another primer set. These results indicate that expression of *BoHsp70* and *BoSCL13* (B_1044548) can distinguish heat shock-tolerant lines for cabbage breeding.

### 2.8. Comparison of Secondary Metabolite Profiling between HTCL “HO” and HSCL “JK”

**Figure 7:**
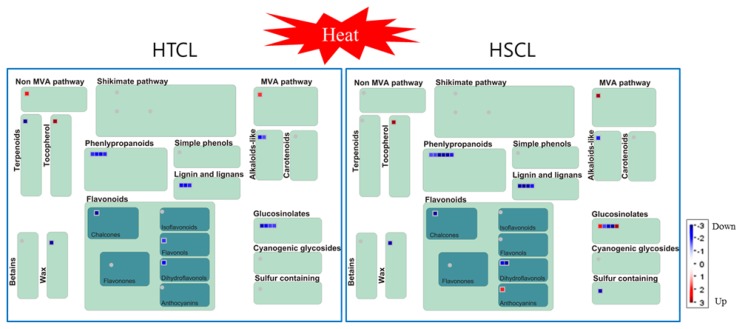
Molecular secondary metabolism events in heat stress-tolerant and heat-sensitive cabbage inbred lines in the heat stress condition. Differentially expressed genes are depicted by MapMan format (ver. 3. 5. 1), in which the square represents a gene. Red and blue colors depict up- and down-regulated genes, respectively. Gene data were examined by MapMan analysis, which is provided in Table 3. HO, heat stress-tolerant cabbage inbred line; JK, heat stress-sensitive cabbage inbred line.

Environmental stress causes exchange of secondary metabolites as an adaptation for overcoming the constraints of stress. We compared gene expression profiles of secondary metabolites between HTCL and HSCL at the HS condition with a MapMan program. The MapMan analysis showed that differentially expressed genes in both cabbage lines were associated with secondary metabolites, such as alkaloid-like molecules, flavonoids, glucosinolates, non-mevalonate, phenylpropanoid, sulfur-containing and terpenoids (Figure 7). Specific genes in the HTCL were involved in non-mevalonate, terpenoid and flavonol pathways. Specific genes in the HSCL were encoded anthocyanins, glucosinolates and sulfur-containing molecules. Other genes showed the same expression patterns. Interestingly, three upregulated genes in the HSCL were in the anthocyanin and glucosinolate pathways (Table 3). Thus, several genes involved in the secondary metabolite pathway were differentially regulated in HTCL and HSCL at the heat stress condition. Further studies are needed to understand the mechanism.

## 3. Discussion

Cabbage shows the most prominent head formation compared to other *Brassicaceae* plants. The ability to form heads at a high temperature is recognized as a major trait identifying high temperature-resistant varieties of cabbage. HTCLs in this study showed much better head formation compared to HSCLs with green house cultivation at a temporary summer temperature over 40 degrees (Figure 1a,b). Lines selected for heat tolerance showed similar differences in their responses to high temperature *in vitro* as young plants (Figure 4a,b). Differential gene expression was compared between HTCL, “HO” and HSCL, “JK”, young plants at HS condition. Many genes were up- and down-regulated in both HTCL and HSCL. GO analysis classified genes that were upregulated as involved in stimulus response, signaling, immune response and transcriptional regulation activity. Downregulated genes were classified as being involved in metabolic processes, cellular processes, developmental processes and binding. These results did not differ significantly from other results showing changes in cellular metabolism at the stress condition (Figure 2a,b). Expression of most Hsps is increased if cells are exposed to elevated temperature or other stressful conditions, although some Hsps are expressed even in non-stressful conditions [42]. Further variations in Hsp expression have been observed even within the same species in thermally-contrasting habitats. Variations in Hsp production often correlates with heat tolerance. For example, sHsps are differentially expressed in distinct varieties of common beans according to their heat stress tolerance [43]. Potatoes exhibited genotypic differences in thermotolerance and heat shock responses in four cultivars, two of which were heat-tolerant cultivars and two heat-sensitive cultivars [44]. Two distinct inbred lines of Chinese cabbage, which have different geographic origins and different responses to temperature and vernalization, had different expression levels of some Hsps and Hsfs in short-term HS stress conditions [38]. Variations in expression of Hsps were also observed between HTCLs and HSCLs and within HTCLs and HSCLs (Figures 5 and 6; Tables 1 and 2). Nevertheless, expression levels of many Hsp genes (about 40) were increased by more than four-fold at HS, suggesting that Hsp genes play critical roles in adapting to heat stress. *BoHsp70* was increased at the HS condition, as were other Hsps. However, expression of *BoHsp70* was increased by approximately three- to four-fold in all HTCL lines, whereas expression was increased by 0.5-fold or equivalent in HSCLs. Expression of *BoHsp70* was significantly different between HTCLs and HSCLs. In addition, qRT-PCR analysis with other primers reconfirmed differential expression of *BoHsp70* (Figure 6a). Other *BoHsp70* family genes were not genetic markers that distinguished HTCLs from HSCLs, due to a high degree of variation between each line and unclear differences between HTCLs and HSCLs (Figure 6a). The *Arabidopsis* ortholog of *BoHsp70*, *AtHsp70-4* or *AtHsp70* (At3g12580) is a member of the Hsp70 family and is localized to the cytoplasm. Expression of *AtHsp70* is constitutive and highly heat-inducible [45,46].

The GRAS plant-specific transcription factor family has a major role in the developmental process [47], but its functions in the heat stress response have not been verified. The GRAS name was derived from the first three members to be cloned, which were GAI, RGA and SCR [48–52]. The GRAS protein structure consists of divergent *N-*terminal regions and VHIID, PFYRE and SAW motifs. GRAS proteins share sequence homology at the *C-*terminal regions [52]. The *Arabidopsis* and rice genomes encode more than 33 and 60 GRAS genes, respectively [53–57]. SCL13 has been identified as a PAT (phytochrome A signal transduction) 1 group GRAS gene and plays a role in red light signal transduction as a positive regulator [58]. Suppression of *SCL13* transgenic plants had reduced sensitivity to red light, suggesting that SCL13 functions in hypocotyls elongation during de-etiolation. However, other potential biological functions have not yet been studied, including heat stress tolerance. In this study, expression levels of *BoSCL13* were increased with HS in all cabbage lines (Figure 5b). Furthermore, the ortholog of *AtSCL13* (At4g17230) was also increased by heat shock at an early time point after heat treatment (data not shown). Therefore, SCL13 may be involved in heat tolerance. *BoSCL13* (B_1044548) also showed distinctive differences in HTCLs and HSCLs. There was a three-fold increase in *BoSCL13* (B_1044548) in HTCLs compared to HSCLs, even though qRT-PCR analyses were conducted with two different primers at the HS condition (Figures 5b and 6b). In addition, the other *BoSCL13* (X_1024979) gene did not show a differential of gene expression between HTCLs and HSCLs at the HS condition, suggesting that the *BoSCL13* (B_1044548) is a unique candidate gene for discriminating heat shock tolerance in cabbage breeding. In summary, these results suggested that the *BoHsp70* and *BoSCL13* (B_1044548) genes might be related to heat tolerance and/or proper head formation of cabbage at high temperature. Further, these data suggested that increased expression of these genes might be markers that distinguish heat-tolerant cabbage lines. Future studies will examine the specific roles of these proteins in heat tolerance and/or heat response.

## 4. Experimental Procedures

### 4.1. Plant Materials

Eight inbred lines of cabbage (*Brassica oleracea* L) were selected as HTCL or HSCL, based on their abilities to form heads after one summer season in a vinyl greenhouse. The “HO”, “KK”, “RK” and “401” showed normal head formation, whereas the “EB”, “JK”, “NB” and “402” showed poor head formation. The eight *B. oleracea* genotypes were “HO”, “KK”, “RK”, “401”, “EB”, “JK”, “NB” and “402” and were obtained from the Asia Seed Company (Gyeonggi-Do, Korea). The lines, “HO”, “KK”, “RK” and “401”, were determined to be HTCLs, and “EB”, “JK”, “NB” and “402” were HSCLs.

### 4.2. Plant Growth Conditions

Field experiments were conducted at the Gwangju location in Gyeonggi-Do Province, Korea. Eight lines were grown from May to October to select for heat tolerance. Plants were sown and grown during the last days of May and first days of June with the distance between plants of 60 cm in two rows of 40 cm distance at 25 days post-emergence. After the summer season, heat tolerance was identified by head formation in each inbred line. Seeds were surface-sterilized with 70% ethanol for 5 minutes (min) and with 10% chlorax for 30 min in the growth chamber. Seeds were then rinsed with sterilized distilled water five times for 5 min. Seeds were germinated on Murashige and Skoog (MS) media containing 1% sucrose for 7 to 10 days in an 8 × 150 mm petri dish. Seeds of soil-grown plants were directly germinated in sterilized soil and grown for 2 to 4 weeks. Plants were maintained at controlled growth conditions of 16-hour day/8-hour night cycles at 24 °C with 150 μE m^−2^s^−1^ in a growth chamber.

### 4.3. Heat Treatments

Two-week-old, young soil-grown plants were heat-shocked at 42 °C for 24 h and then transferred to 24 °C for recovery and phenotypic analyses. One-week-old plate-grown sterilized seedlings were heat-shocked in petri dishes at 42 °C for 5 h in an incubator and then recovered at 24 °C in a growth chamber. Phenotypic characteristics appeared approximately 3 days after transfer to room temperature. For Genechip analysis, 2-week-old, young soil-grown HTCL, “HO”, and HSCL, “JK”, plants were heat-shocked at 42 °C for 2 h (HS) or continuously grown at 24 °C (NS).

### 4.4. Genechip Analysis

Cyanine 3-labeled and cyanine 5-labeled cRNA were produced from total RNA with the Low Input Quick Amp Labeling Kit (Agilent Technology, Santa Clara, CA, USA), according to the manufacturer’s instructions. The amounts and qualities of labeled cRNAs were assessed with a NanoDrop ND-1000 spectrophotometer and an Agilent Bioanalyzer. Expression profile analysis was performed on a Brassica 105k oligo microarray (2 × 105k format; Agilent Technologies, SurePrint™ Technology) [56]. Hybridization images were analyzed on an Agilent DNA microarray Scanner (Agilent Technology), and quantification was performed with Agilent Feature Extraction software 10.7 (Agilent Technology). The average fluorescence intensity for each spot was calculated and local background was subtracted. Data normalization and selection of genes showing changes in expression were performed with GeneSpringGX 7.3.1 (Agilent Technology). Genes were filtered and flag-out genes were removed. LOWESS normalization of data was performed [59]. Spots that did not meet the minimum signal intensity were removed. The remaining signal data were analyzed with the student’s *t*-test (*p*-value ≤ 0.05), assuming normality, but not equal variances, with a Benjamini-Hochberg correction for multiple comparisons and was calculated as the log_2_-transformed signal ratio. The GeneSpring cross-gene error model, which was considered present in two biological replicate experiments and was determined to have a 2-fold change, was active during this study.

### 4.5. Identifying Biological Functions of Differentially Expressed Genes

BLASTN hits were found in *Arabidopsis* for 44,881 (HTCL, “HO”) and 47,352 (HSCL, “JK”) of the 103,748 *Brassica* unigenes [56]. The top *Arabidopsis* hit corresponding to each *Brassica* unigene was analyzed for function. Functional classification was carried out with the agriGO web-based GO analysis toolkit [45]. *Arabidopsis* genes were put into the Singular Enrichment Analysis (SEA) with the suggested reference background (TAIR release 10). The Fisher’s Exact Test with Yekutieli-adjusted *p*-values was employed in the SEA analysis with the “complete GO” ontology.

### 4.6. Quantitative Real-Time PCR (qRT-PCR) Analysis

Total RNA was isolated from leaf tissue with RNAiso Plus (TaKaRa Bio Inc., Otsu, Japan). The cDNAs were synthesized with M-MLV reverse transcriptase and oligo (dT) primer in a 20 μL volume, according to the manufacturer’s instructions (Invitrogen, Grand Island, NY, USA). Quantitative PCR was performed in 10 μL reactions with gene-specific primers (Table S1), 1 μL of cDNA as template and SYBR Premix Ex Taq (TaKaRa Bio Inc., Otsu, Japan). Reactions were performed on the CFX96 Real-Time PCR system (BioRad, Hercules, CA, USA). The thermal profile for qPCR was 3 min at 95 °C, followed by 40 cycles each of 95 °C for 20 s, 60 °C for 20 s and 72 °C for 20 s. Primer specificity and formation of primer-dimers were monitored by dissociation curve analysis. The expression level of *Brassica oleracea actin1* (*BoActin1*) served as an internal standard for normalization of cDNA template quantity and was measured with actin-specific primers (Table S1). The qRT-PCR reactions were performed as three biological and three technical repeat. Furthermore, we showed a representative data in the figures, because the different biological experiments showed a similar expression pattern. For the qRT-PCR analysis, a pooling of 5 young plants in each inbred line were used in the figures.

### 4.7. Heat Shock-Related Gene Expression

First-strand cDNA products were analyzed by qRT-PCR with each set of heat responsive gene-specific primers. Expression of the *BoActin1* gene was the internal control. Primers specific to *B. oleracea* were designed with sequences obtained from the NCBI/TAIR/CsCG *B. oleracea* EST internal database (http://cgc.kribb.re.kr:8080/brdb/; in preparation). Primers were designed to amplify conserved regions of each gene, which was determined by alignments with *Arabidopsis* orthologs. Gene-specific primers are described in the supplemental table (Table S1). The identities of *B. oleracea* heat-responsive genes were confirmed by sequence analysis.

### 4.8. MapMan Analysis

Differential gene expression changes between HTLC and HSCL in the HS condition were constructed with MapMan (v. 3.5.1. R2), which was generated by MapCave analysis with gene identifiers corresponding to 105k-probe sets of the Brassica DNA chip. Data were organized and displayed at specific locations on diagrams of secondary metabolism based on the SCAVENGER and IMAGEANNOTATOR modules of the MAPMAN tool [60].

## 5. Conclusions

Detailed information for all cabbage Hsfs and Hsps is not yet available. However, constitutive or strong expression of several heat stress-related genes may contribute to proper cabbage head formation at normal growing temperatures or high-stress temperatures. Expression levels of these genes may serve as markers for faster and easier HTCL selection in cabbage breeding.

## Supplementary Information



## Figures and Tables

**Table 1:** Differentially expressed heat shock protein genes under heat stress in inbred line HO and JK.

Classification	Probe name a	TAIR_ID b	Description	HO (HS/NS) c	JK (HS/NS) d
	
				FC e	FC
Up-regulated					
hsp100	B_1084558	AT1G74310	Clp/Hsp100 (Heat shock protein 101)	7.90	6.90
	B_1078483	AT1G74310	Clp/Hsp100 (Heat shock protein 101)	6.07	5.96
	X_1024246	AT1G74310	Clp/Hsp100 (Heat shock protein 101)	3.00	2.77
	B_1047985	AT1G74310	Clp/Hsp100 (Heat shock protein 101)	7.05	7.12
	B_1045948	AT5G15450	ClpB heat shock protein-like	4.20	5.17
	B_1026871	AT5G15450	ClpB heat shock protein-like	3.81	4.10
hsp70	B_1058880	AT1G16030	HSP70b (Heat shock protein Hsp70)	5.28	5.94
	B_1067101	AT2G32120	HSP70T-2 (70 kD heat shock protein)	3.61	4.11
	B_1048388	AT3G12580	HSP70 (70 kDa heat shock protein)	7.11	7.08
	B_1047992	AT3G12580	HSP70 (Heat shock protein 70)	6.51	6.75
	B_1071737	AT4G24280	CPHSC70-1 (Chloroplast HSP70)	2.57	2.52
	B_1020203	AT4G24280	CPHSC70-1 (Hsp 70-like protein)	2.47	2.09
	X_1034152	AT5G02500	HSC70-1 (Heat shock cognate 70 kDa protein 1)	3.29	2.33
	B_1085663	AT5G02500	HSC70-1 (Heat shock cognate protein 70)	2.25	-
	B_1051923	AT5G09590	HSC70-5 (Heat shock protein 70)	4.58	4.24
	B_1002974	AT5G09590	HSC70-5 (Heat shock protein 70)	3.13	2.69
	B_1001993	AT5G09590	HSC70-5 (Heat shock protein 70)	2.63	2.44
hsp90	B_1046678	AT5G52640	Heat shock protein 81-1	5.53	5.88
	B_1060380	AT5G56030	Heat shock protein 81-2	4.41	4.32
shsp	B_1022192	AT3G17350	17.5 kDa class I heat shock protein	3.58	3.36
	X_1001732	AT5G12020	17.6 kDa class II heat shock protein	6.51	6.72
	X_1077107	AT5G12020	17.6 kDa class II heat shock protein	6.29	6.18
	X_1084898	AT5G12030	17.6 kDa class II heat shock protein	5.61	6.11
	B_1055398	AT5G59720	Hsp18.2 (Heat shock protein 18)	7.72	8.61
	X_1064115	AT5G59720	Hsp18.2 (Heat shock protein 18)	7.12	6.92
	B_1071667	AT5G59720	Hsp18.2 (Heat shock protein 18)	6.53	8.62
	B_1052943	AT4G27670	Putative heat shock protein 21	8.50	8.66
	B_1001981	AT4G10250	22.0 kDa class IV heat shock protein precursor	6.67	6.78
	B_1047320	AT4G10250	AtHSP22.0 precursor	9.17	9.08
	B_1056315	AT4G25200	Heat shock 22 kDa protein, mitochondrial precursor	6.57	6.66
	B_1015145	AT5G51440	Mitochondrial heat shock 22 kd protein-like	2.29	2.13
	B_1050062	AT5G51440	Mitochondrial heat shock 22 kd protein-like	2.13	2.09
	B_1082795	AT2G19310	Putative small heat shock protein	4.79	4.50
	B_1033013	AT2G29500	Cytosolic class I small heat shock protein 3B	5.11	5.00
	B_1055192	AT2G29500	Putative small heat shock protein	3.26	2.85
unclassified	B_1081966	AT1G54050	Heat-shock protein, putative	5.74	6.07
	B_1066881	AT2G20560	Putative heat shock protein	6.78	6.45
	B_1013334	AT2G35330	Putative heat shock protein	2.65	3.34

Down-regulated					
hsp70	B_1050323	AT4G37910	Heat shock protein 70 like protein	−3.07	−3.31
	B_1054152	AT4G37910	Heat shock protein 70 like protein	−3.05	−2.98

* Differential expression with fold-change values of ≥2 and ≤−2 at *p* < 0.05, respectively;

a Probe Name is the probe number of cabbage microarray;

b TAIR_ID showing the best hit when BlastN was performed against the TAIR release 10 database;

c HO (HS/NS) showing the gene expression of the HO cabbage line comparing heat stress with no heat stress condition;

d JK(HS/NS) showing the gene expression of the JK cabbage line comparing heat stress with no heat stress condition;

e FC is fold change values (log_2_).

**Table 2:** Differentially expressed heat stress transcription factors genes under heat stress in inbred line HO and JK.

Classification	Probe name a	TAIR_ID b	Description	HO (HS/NS) c	JK (HS/NS) d
	
				FC e	FC
Upregulated					
	B_1065889	AT2G26150	HsfA2 (Heat stress transcription factor A-2)	6.39	6.09
	B_1018750	AT2G26150	HsfA2 (Heat stress transcription factor A-2)	6.04	6.37
	B_1004068	AT2G26150	HsfA2 (Heat stress transcription factor A-2)	5.52	5.01
	B_1019012	AT3G51910	HsfA7a (Heat stress transcription factor A-7a)	2.99	4.62
	B_1024396	AT3G51910	HsfA7a (Heat stress transcription factor A-7a)	5.79	5.50
	B_1023370	AT3G51910	HsfA7a (Heat stress transcription factor A-7a)	4.49	5.31
	X_1078901	AT3G51910	HsfA7a (Heat stress transcription factor A-7a)	4.53	4.83
	X_1062243	AT4G36990	HsfB1 (Heat stress transcription factor B-1)	4.28	3.79
	B_1066120	AT4G11660	HsfB2b (Heat stress transcription factor B-2b)	3.62	5.42
	B_1050506	AT4G11660	HsfB2b (Heat stress transcription factor B-2b)	4.92	5.16
	X_1043486	AT3G24520	HsfC1 (Heat stress transcription factor C-1)	3.84	3.69

* Differential expression with fold-change values of ≥2 (*p* < 0.05);

a Probe Name is the probe number of cabbage microarray;

b TAIR_ID showing the best hit when BlastN was performed against the TAIR release 10 database;

c HO (HS/NS) showing the gene expression of the HO cabbage line comparing the heat stress with the no heat stress condition;

d JK (HS/NS) showing the gene expression of the JK cabbage line comparing the heat stress with no heat stress condition;

e FC is fold change values (log_2_).

**Table 3:** Differentially expressed genes were involved in secondary metabolisms in the HO and JK inbred line after heat stress treatment.

BinCode a	BinName b	At Id c	Gene description	Probe name d		Fold change	
	
				HO e	JK f	HO	JK
16.1.1.10	non-mevalonate pathway. geranylgeranyl pyrophosphate synthase	at4g36810	GGPS1 (GERANYLGERANYL PYROPHOSPHATE SYNTHASE 1)	B_1036920	-	2.35	-

16.1.2.1	isoprenoids. mevalonate pathway. acetyl-CoA *C*-acyltransferase	at5g47720	acetyl-CoA *C*-acyltransferase, putative	B_1048161	B_1048161	2.18	3.88
				B_1000524	B_1000524	2.17	3.63
				-	B_1056048	-	3.64

16.1.5	isoprenoids. terpenoids	at1g78970	LUP1 (LUPEOL SYNTHASE 1)	B_1022598	-	−3.36	-

16.1.3.1	isoprenoids. tocopherol biosynthesis. hydroxyphenylpyruvate dioxygenase	at1g06570	PDS1 (PHYTOENE DESATURATION 1)	B_1070419	B_1070419	3.23	4.69

16.2	phenylpropanoids	at1g77520	*O*-methyltransferase family 2 protein	B_1076342	B_1076342	−2.20	−2.04
		at5g07870	transferase family protein	-	B_1070123	-	−2.09

16.2.1.3	phenylpropanoids. lignin biosynthesis.4CL	at1g65060	4CL3 (4-coumarate-CoA ligase)	-	B_1058738	-	−3.72
				-	B_1064185	-	−2.22
				-	B_1028095	-	−2.19

16.2.1.4	phenylpropanoids. lignin biosynthesis. HCT	at5g48930	HCT (HYDROXYCINNAMOYL-COA SHIKIMATE/QUINATE HYDROXYCINNAMOYL TRANSFERASE)	-	B_1072263	-	−3.63

16.2.1.6	phenylpropanoids. lignin biosynthesis. CCoAOMT	at1g24735	*O*-methyltransferase	B_1079441	-	−2.39	-

16.2.1.9	phenylpropanoids. lignin biosynthesis. COMT	at5g54160	ATOMT1 (O-METHYLTRANSFERASE1)	X_1039499	X_1039499	−2.54	−3.09

16.2.1.10	phenylpropanoids. lignin biosynthesis. CAD	at2g21730	CAD2 (CINNAMYL ALCOHOL DEHYDROGENASE HOMOLOG 2)	B_1059602	B_1046994	−2.25	−2.15
				B_1046994	B_1059602	−2.15	−2.32

16.4.1	N misc. alkaloid-like	at4g28680	TYRDC1 (tyrosine decarboxylase, putative)	X_1033105	-	−2.39	-
				B_1077763	-	−2.31	-
		at5g22020	strictosidine synthase family protein	B_1063326	-	−2.03	-
		at3g57010	strictosidine synthase family protein	-	X_1052064	-	−2.32

16.7	wax	at5g57800	CER3 (ECERIFERUM 3)	B_1062054	B_1062054	−3.11	−2.90
				B_1058964	B_1058964	−2.39	−2.36
				B_1036813	-	−2.30	-
				X_1062146	X_1062146	−2.28	−2.72
				X_1073290	X_1073290	−2.26	−2.63

16.8.1.21	flavonoids. anthocyanins. anthocyanin 5-aromatic acyltransferase	at3g29670	transferase family protein	-	B_1000057	-	2.27

16.8.2.1	flavonoids. chalcones. naringenin-chalcone synthase	at5g13930	TT4 (TRANSPARENT TESTA 4)	B_1078676	B_1078676	−4.08	−3.68
				B_1049448	B_1049448	−3.28	−3.22
				B_1083997	B_1083997	−3.16	−3.62

16.8.3	flavonoids. dihydroflavonols	at5g54010	glycosyltransferase family protein	B_1048955	B_1048955	−2.43	−2.51

16.8.3.1	flavonoids. dihydroflavonols. dihydroflavonol 4-reductase	at5g42800	DFR (DIHYDROFLAVONOL 4-REDUCTASE)	-	B_1047840	-	−3.85

16.8.4.1	flavonoids. flavonols. flavonol synthase (FLS)	at5g08640	FLS (FLAVONOL SYNTHASE)	B_1067180	-	−2.17	-

16.5.1.1.1.2	sulfur-containing. glucosinolates. synthesis. aliphatic. methylthioalkylmalate synthase (MAM)	at5g23010	MAM1 (METHYLTHIOALKYLMALATE SYNTHASE 1)	-	B_1017825	-	2.38

16.5.1.1.3.1	sulfur-containing. glucosinolates. synthesis. indole. CYP79B2 monooxygenase	at4g39950	CYP79B2	B_1071664	B_1071664	−2.84	−2.06

16.5.1.1.4.1	sulfur-containing. glucosinolates. synthesis. shared. CYP83B1 phenylacetaldoxime monooxygenase	at4g31500	CYP83B1 (CYTOCHROME P450 MONOOXYGENASE 83B1)	B_1082264	B_1082264	−2.07	−2.58
				-	B_1046742	-	−2.78

16.5.1.3.1.1	sulfur-containing. glucosinolates. degradation. myrosinase. TGG	at5g26000	TGG1 (THIOGLUCOSIDE GLUCOHYDROLASE 1)	B_1057241	B_1057241	−2.11	−3.30

16.5.1.3.2.1	sulfur-containing. glucosinolates. degradation. nitrile-specifier protein. epithio-specifier protein	at1g54040	ESP (EPITHIOSPECIFIER PROTEIN)	X_1061657	-	−2.10	-

16.5.1.3.2	sulfur-containing. glucosinolates. degradation. nitrile-specifier protein	at5g48180	NSP5 (NITRILE SPECIFIER PROTEIN 5)	-	B_1062808	-	3.25

16.5.99.1	sulfur-containing. misc. alliinase	at4g24670	TAR2 (TRYPTOPHAN AMINOTRANSFERASE RELATED 2)	-	B_1048365	-	−2.85

* Differential expression with fold-change values of ≥2, ≤−2 (*p* < 0.05), respectively;

a BinCode is the number that is assigned in the measured parameters to hierarchical categories by MapMan;

b BinName is information about the BinCode;

c At ID showing the best hit when BlastN was performed against the TAIR release 10 database;

d Probe Name is the probe number of the cabbage microarray;

e HO represent heat tolerant cabbage lines of the heat stress condition;

f JK represent heat sensitive cabbage lines of the heat stress condition.
