# Supplementary material for: Use of Heat Stress Responsive Gene Expression Levels for Early Selection of Heat Tolerant Cabbage (Brassica oleracea L.)
Source: Int J Mol Sci. 2013 Jun 4;14(6):11871–94. doi: 10.3390/ijms140611871 (PMC3709761; doi:10.3390/ijms140611871)
Supplement: Supplementary file 1 [file ijms-14-11871-s001.pdf]

Supplementary Information

Table S1. Primer pairs used for qRT-PCR analysis of *Brassica oleracea* gene expression.

Gene name	EST database	Primer sequences (F, forward; R, reverse [5'−3'])	
B_1087048_ Splicing factor	DK544951	F: gggacttgaggaaaggcgaagc	R: gaatcctcctagacgtcgtgtccc
B_1044548_SCL13	DK561192	F: ccgaaactcgtgacgctagtggagc	R: gcggaacatgtagccatgggtctcc
B_1048388_Hsp70	DK546191	F: gggaaggctgtccaaggaagag	R: ccatgccaccagctccacccatatc
B_1029242_Myb t.f	EV097689	F: gagatgtagagtgggagggag	R: cttgaaggccacaaactggcc
B_1036869_Pectate lyase18	EV178268	F: tgccataatgggctccacggc	R: ccagaggatctccagttccagc
B_1081233_ATP synthase	EV104543	F: cgactgcctcttcgattccgacg	R: gcggcagaagcaagtttctgcg
B_1078483_Clp/Hsp100	CD837787	F: ctggaagcagcaaggagcaactgg	R: cacactctcatcctctgggtcgctag
B_1071667_Hsp18.2	CV830558	F: cgaaggtacatctgccttctt	R: ggaatgagaagctcagttatg
X_1078901_HsfA7a	ES902269	F: ctgctgtcgatggagctggcaag	R: ctccagagccagagcctcaagttc
B_1058749_HsfA1a	EE531559	F: gagactcactgaggtgc	R: gtcgctgctctctagtgttgtctc
B_1065889_HsfA2	EV112465	F: gtggaagtcgggcaatacgg	R: gccccaatccaacggtgaac
B_1066120_HsfB2b	DK469732	F: gagattcgtcacggactctg	R: gaacagcagcagcgacggtaac
B_1058880_Hsp70b	AM389634	F: gatgagacgttagaggggat	R:catcacagtatcaaaacacctc
B_1067101_Hsp70T-2	EE408040	F: ggatatcgatgcttcgaatgc	R: acgcatacacacattgtcac
B_1001300_Hsp70T-2	AM395056	F: aggaagctctagaagatcaaatc	R: agagtagtataacagctgtcg
B_1085663_Hsc70-1	ES920448	F: gctgagaagtacaagtctgagga	R: gctccttgatacatcttagcaat
X_1034152_Hsc70-1	EV146186	F: agtcgacttcctcaatcttggg	R: atgtgcgttaggtcactcatg
B_1051923_Hsc70-5	EE443209	F: tgtctggtggaggttctgca	R: cattaagcttcgttcaccaca
B_1020203_Cphsc70-1	ES908982	F: cggtggaattggaatcgctactta	R: cctactaccaataaacctcttc
X_1024979_SCL13	ES999914	F: gagggtgagaatgatgatgg	R: acaggctggcagatgtgtgttg
B_1024396_HsfA7a	ES985248	F: aagtgtcgagggagaagaaacg	R: gggtttccagaactcgatga
B_1046678_Hsp81-1	AM060916	F: gtatccacgaggacagtcagaac	R: agttttaacccttcctttgttgc
B_1055398_Hsp18	EE470714	F: gggatccatttgaaggactctta	R: tttattccacacaccaaatcctc
X_1064115_Hsp18	CX279910	F: ttttcgatccgttctcactagac	R: aacacgctttttattccacacac
X_1001732_Hsp17.6	BG543222	F: aatctcaatcctcgaagacatgc	R: gattttcagcagtgaacttcctca
B_1056315_Hsp22	EE421035	F: tattttcacagacgtgttcgatcc	R: gtttcagttgatttcgatctgacg
B_1066881_DnaJ	EV017880	F: aaaggaaccaccaagaagatgaag	R: ttacagatccgaccaaacaggata
B_1046678_HSP81	DY027487	F: tacctcagtttcgtgaaaggtgt	R: cgaatgacttcttttctcttcc
B_1001981_Hsp22	BQ790750	F: tttcagagtcttggagcaaatcc	R: cgttctcaagcttggccttaata
B_1047320_Hsp22	EV045072	F: ggagaatcgagtcctctcagtca	R: gatctgttgcaagggattacacc
BoACT1	AF044573(cds)	F: cagtgtctggatcggtggttcc	R: gaggcatacatcaattcgatcac

© 2013 by the authors; licensee MDPI, Basel, Switzerland. This article is an open access article distributed under the terms and conditions of the Creative Commons Attribution license (http://creativecommons.org/licenses/by/3.0/).
